# Basic Fibroblast Growth Factor Attenuates Injury in Myocardial Infarction by Enhancing Hypoxia-Inducible Factor-1 Alpha Accumulation

**DOI:** 10.3389/fphar.2020.01193

**Published:** 2020-08-07

**Authors:** Zhiheng Rao, Danping Shen, Jiahui Chen, Lushen Jin, Xueping Wu, Ming Chen, Lei Li, Maoping Chu, Jiafeng Lin

**Affiliations:** ^1^ Department of Cardiology, The Second Affiliated Hospital of Wenzhou Medical University, Wenzhou, China; ^2^ Department of Clinical Medicine, The Second School of Wenzhou Medical University, Wenzhou, China; ^3^ Department of Pediatric Cardiology, The Second Affiliated Hospital and Yuying Children’s Hospital of Wenzhou Medical University, Wenzhou, China; ^4^ Department of Cardiology, Taishun People’s Hospital, Wenzhou, China; ^5^ Department of Clinical Medicine, The First School of Wenzhou Medical University, Wenzhou, China

**Keywords:** hypoxia-inducible factor-1 alpha, basic fibroblast growth factor (FGF2), ischemic cardiomyopathy, angiogenesis, antiapoptosis

## Abstract

**Background:**

The combination of antiapoptotic and angiogenic actions may represent a pharmacotherapeutic strategy for the treatment of myocardial infarction. Fibroblast growth factor (FGF) is expressed in various cell types including endothelial and muscle cells and promotes their survival, migration, and proliferation.

**Methods and Results:**

Myocardial microvascular endothelial cells were divided into four treatment groups, the sham, hypoxia, basic FGF (bFGF), and bFGF plus 2-methoxyestradiol groups, and subjected to *in vitro* apoptotic analysis and Matrigel assays. An *in vivo* model of myocardial infarction was established by ligaturing the left coronary artery of mice in the four treatment groups. Cardiac performance, myocardial injury, endothelial cell angiogenesis, and myocardial apoptosis were assessed. bFGF administration after myocardial infarction improved cardiac function and cell viability, attenuated myocardial injury and apoptosis, and enhanced angiogenesis. Western blotting of HIF-1α, p-AKT, VEGF, p53, BAX, and Bcl-2 showed that bFGF increased HIF-1α, p-AKT, VEGF, and Bcl-2 and decreased BAX protein levels.

**Conclusion:**

The results of the present study indicated that bFGF attenuates myocardial injury by inhibiting apoptosis and promoting angiogenesis *via* a novel HIF-1α-mediated mechanism and a potential utility of bFGF in protecting against myocardial infarction.

## Introduction

Coronary atherosclerosis remains the leading cause of poor clinical outcomes associated with heart disease such as myocardial infarction (MI) and has a high mortality rate in both China and worldwide (Gaziano, 2005; Nabel and Braunwald, 2012; Fordyce et al., 2015; Mozaffarian et al., 2015). A number of different treatment methods have been developed based on the pathogenesis (Gersh, 2008; Bagai et al., 2014), such as ventricular remodeling and reduction of the infarcted area during the later stages of MI using pharmacological agents (Fihn et al., 2012).

The fibroblast growth factor (FGF) family consists of 22 members in humans and mice (Ornitz and Itoh, 2015). Most FGFs play roles *via* autocrine/paracrine signaling in health, development, and disease in various organs including the heart (Ornitz and Marie, 2015). They have been suggested to play roles in the proliferation, migration, differentiation, and survival of many types of cells, including the endothelial cells (Palmen et al., 2004). Among the members of the FGF family, basic FGF (bFGF or FGF2) has been implicated as an angiogenic factor secreted upon contraction of the myocardium, which shows cardioprotective effects in animal models (House et al., 2003), and has been reported to be beneficial for ischemic injury in some clinical trials (Laham et al., 1999; Simons, 2002). The cardioprotective effect of bFGF in ischemic injury may involve neovascularization (House et al., 2003).

The effects of bFGF are mediated by FGF receptors (FGFR1–4), which are cell-surface receptors of the tyrosine kinase family (Szebenyi et al., 1998). Among the FGFRs, FGFR1 is highly expressed and has a prominent role in adult cardiomyocytes (Jin et al., 1994). Activated FGFRs phosphorylate or recruit downstream signaling molecules that activate several major intracellular signaling pathways, such as the MAPK, PKC, Src-associated, and PI3K/Akt pathways (Detillieux et al., 2003), leading to cell differentiation, proliferation, and survival.

However, how bFGF reduces the injury associated with myocardial infarction has not been clearly understood. It was reported that the cardioprotective effects of bFGF may be related to angiogenesis or antiapoptotsis *via* the accumulation of HIF-1α in the infarcted area. Activated HIF-1α plays an important role in the adaptive responses of tumor cells to changes in oxygen levels *via* transcriptional activation of downstream genes that regulate crucial biological processes required for tumor progression and survival, including genes involved in migration, cell proliferation, and angiogenesis (Semenza, 2001; Matsunaga et al., 2009). 2-Methoxyestradiol (2-MeOE2) is the inhibitor of HIF-1α which can block the activation of HIF-1α (Tang et al., 2018).

The present study was performed to investigate protection of the myocardium by bFGF and possible mechanism *via* promotion of both antiapoptotic effects and endothelial cell angiogenesis.

## Materials and Methods

### Reagents and Antibodies

All reagents used in the study were obtained from commercial sources. DMEM and FBS were purchased from Invitrogen (Carlsbad, CA, United States) and bFGF from Grost Biotechnology (Zhejiang, China). Anti-HIF-1α and anti-CD31 antibodies were purchased from Abcam (Cambridge, United Kingdom, United States); anti-Bcl-2, anti-BAX, anti-AKT, anti-phospho-AKT, and anti-p53 antibodies from Cell Signaling Technology (Danvers, MA, United States); anti-VEGF (A-20) antibody from Santa Cruz Biotechnology (Santa Cruz, CA, United States); and anti-alpha-actinin-1 antibody from R&D Systems (Minneapolis, MN, United States). 2-Methoxyestradiol (2-MeOE2) was purchased from Selleck (Selleck, Houston, TX, United States). Alexa Fluor 488 and 594 were purchased from Yeasen (Shanghai, China). Matrigel was purchased from BD Biosciences (Bedford, MA, United States).

This study was reviewed and approved by the Ethics Committee for Experimental Animals of Wenzhou Medical University. Healthy adult female and male Sprague–Dawley rats (~8 weeks old; 220–250 g) and male wild-type C57BL/6J mice (6–8 weeks old; 20–30 g) were used in this study. All animals used in this study were purchased from Slac Laboratory Animal Corporation (Shanghai, China) and housed under controlled humidity (50 ± 10%) and temperature (25°C ± 2°C) and a 12/12-h light/dark cycle.

### Myocardial Infarction Model

The MI mouse model was established using C57BL/6J mice (20–25 g, 6–8 weeks old). The C57BL/6J mice were anesthetized with isoflurane (3.6 mg/h) by orotracheal intubation connected to a ventilator. The heart was exposed after left thoracotomy between the fourth and fifth intercostal spaces. The left anterior descending artery was ligated with a 6-0 silk suture, and mice were randomly grouped to receive the following treatments intraperitoneally: 1) saline (MI group, n=15); 2) bFGF (5 μg/L bFGF in 200 μl PBS, bFGF group, n=15); or 3) bFGF plus 2-MeOE2 (bFGF + 1 mM/L 2-MeOE2 in 200 μl PBS, n=15), once every 2 days for 28 days. The animals were monitored and tissues were collected at 7, 14, and 28 days. As the sham group, another 10 animals received thoracotomy without left coronary artery ligation.

### Culture and Identification of Myocardial Microvascular Endothelial Cells (MMECs)

Sprague–Dawley rats at 8–10 days old were purchased from Slac Laboratory Animal Corporation. The rat hearts were excised in ice-cold PBS, the atrium and right ventricle were removed, and the left ventricular anterior walls were left in place. After washing with PBS to remove blood, the endocardium and epicardium were removed. From the remaining tissue, sections measuring approximately 1 × 1 × 1 mm were cut out and placed in petri dishes pre-wetted with FBS. They were then cultured in an incubator for 6 h. Subsequently, 5 ml complete DMEM supplemented with 10% FBS was added. After 48 h, the tissues were removed, and the culture medium was changed to fresh medium. The cells had a cobblestone mosaic appearance under the microscope. MMECs were identified using the SABC (Strept Avidin-Biotin Complex) method that detects factor VIII; cells with brown coloration of the cytoplasm, indicating a positive reaction, were considered MMECs.

### Assessment of Cardiac Function

On days 7 and 28 after MI, all mice were anesthetized by intraperitoneal injection of 10% chloral hydrate for echocardiography. Echocardiographic parameters were obtained using the Siemens Acuson Sequoia 512C system with a 15 MHz probe. Left ventricular (LV) end systolic and diastolic diameters were calculated in M-mode. LV fraction shortening and LV ejection fraction were then calculated.

### Evaluation of Infarct Size by Pathological Staining

Mice were sacrificed immediately after obtaining echocardiographic measurements. The hearts were arrested, and sections of the myocardium were obtained and stained with Masson’s trichrome. Light microscopy was performed to evaluate staining and morphological changes at 40× and 100× magnifications. Interstitial collagen deposition was measured by Masson’s trichrome staining as the percentage of blue stained tissue. In each section, five random non-overlapping fields were captured using a camera. IPP 6.0 software was used for quantitative analysis of the area of infarction. Stained areas were padded with blue and transformed for OD calibration.

### Matrigel Assay

Prior to Matrigel assays, the MMECs were pretreated with bFGF (100 μM/L) and 2-MeOE2 (1 mM/L) for 12 h. Twelve-well cell culture plates were coated with 500 μl cold Matrigel (4°C) as a base for tube formation. The gels were allowed to set for 30 min in an incubator under 5% CO_2_ at 37°C, and MMECs (6×10^4^ per well) from the different groups were seeded onto the gels and incubated under 5% CO_2_ at 37°C. After a 12-h incubation, the extent of tube formation was observed and recorded by microscopy and then analyzed using NIH Image J software.

### Apoptosis Analysis

MMECs were pretreated with bFGF (100 μM/L) and 2-MeOE2 (1 mM/L) for 12 h in an incubator under 5% CO_2_ at 37°C. Then, cells from each group were placed in a hypoxic incubator (37°C, 1% O_2_, N_2_, and 5% CO_2_) for 12 h, digested with trypsin, washed, centrifuged, and mixed in PBS. The rate of apoptosis was determined by flow cytometry.

### Evaluation of Apoptosis by TUNEL Staining

The frozen tissue was cut into sections of 6–8 μm thick, and terminal deoxynucleotidyl transferase dUTP nick-end labeling (TUNEL) assays were performed using commercial kits (Roche, Mannheim, Germany) to analyze myocardial apoptosis in accordance with the manufacturer’s instructions. The TUNEL-positive cells and nuclei were visualized as blue fluorescence under microscopy. The apoptotic index was calculated as the ratio of TUNEL-positive cardiomyocytes to total nuclei.

### Immunofluorescence

Frozen tissues were cut into sections of 6–8 μm thick, as described above, and incubated for 2 h with primary anti-CD31 antibody (1:300). An Alexa Fluor 594-conjugated secondary antibody (1:500) was used for visualization, with red fluorescence representing vascular endothelial cells and blue fluorescence the nuclei. Images were acquired at 100× magnification by fluorescence microscopy.

### Western Blotting Analysis

For analysis of proteins *in vitro* and *in vivo*, the hearts were homogenized in RIPA buffer (150 mM NaCl, 25 mM Tris-HCl, 1% sodium deoxycholate, 0.1% sodium dodecyl sulfate, and 1% Nonidet P-40) supplemented with phosphatase and protease inhibitors for 30 min at 4°C. The complexes were then centrifuged at 12000 rpm, 4°C, 30 min, and the supernatant was obtained for protein analysis. The protein concentrations were quantified using a BCA kit (Thermo Fisher Scientific, Rockford, IL, United States). Aliquots containing 60 μg total protein were subjected to gel electrophoresis and then transferred to polyvinylidene difluoride membranes. The membranes were blocked with 8% non-fat milk in TBST for 2 h and incubated with the following primary antibodies for 12 h at 4°C: HIF-1α (1:500), p-AKT (1:1000), VEGF (1:1000), AKT (1:1000), BAX (1:1000), p53 (1:1000), Bcl-2 (1:1000), and GAPDH (1:5000). The membranes were washed with TBST three times and incubated with the secondary antibody (1:10000) for 2 h. Signals were visualized using the ChemiDoc™ XRS +Imaging System (Bio-Rad, Hercules, CA, United States). The intensity of immunoreactivity was quantified using NIH Image J software. All experiments were repeated at least 3 times.

### Statistical Analysis

All data are expressed as means ± standard error of the mean of at least three independent experiments. One-way ANOVA analysis of variance was used for multiple comparisons. In all analyses, *P*<0.05 was taken to indicate statistical significance.

## Results

### bFGF Decreased Fibrosis and the Infarcted Area and Preserved LV Function

**Figure 1:**
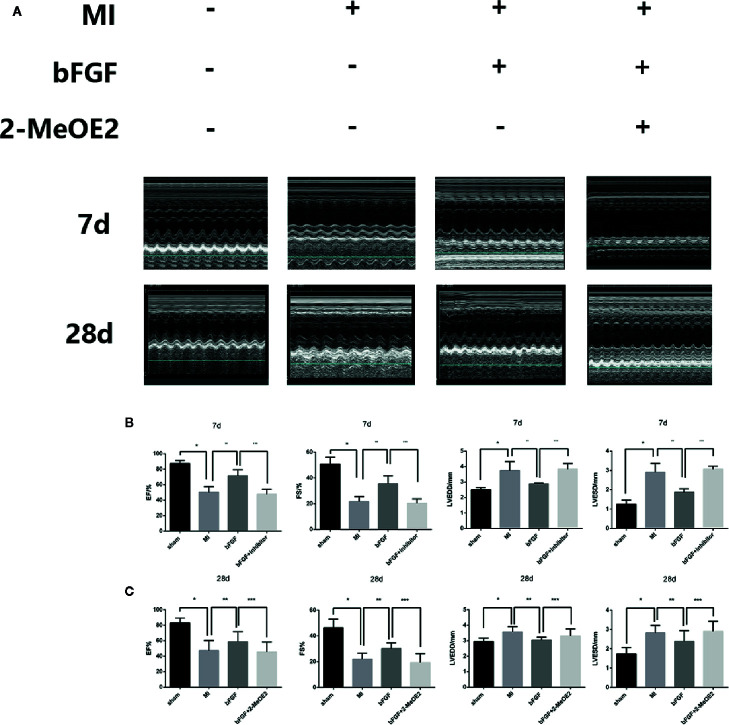
bFGF preserved LV function in mice. **(A)** Effects of bFGF on the infarcted area and LV function. The infarcted areas are echocardiography photos from the sham, MI, bFGF, and bFGF + 2-MeOE2 groups. **(B, C)** EF, FS, LVSD, and LVPW values were compared among the groups. EF, ejection fraction; FS, fraction shortening; LVSD, left ventricular end-systolic diameter; LVPWs, left ventricular posterior wall thickness at end of systole. ^*^ *P*<0.001 vs. sham group. ^**^ *P*<0.01 vs. MI group. ^***^ *P*<0.01 vs. bFGF group (n=5).

**Figure 2:**
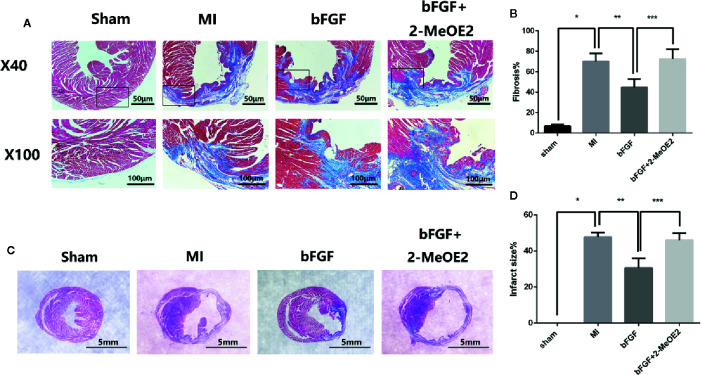
Decrease in the infarcted area and fibrosis in the infarcted zone. **(A, B)** Effects of bFGF on the infarcted area and LV remodeling. Representative images of Masson’s trichrome staining in myocardial sections (5 μm) from the sham, MI, bFGF, and bFGF + 2-MeOE2 groups (40×, 100×). Blue indicates collagen, and red indicates viable myocardium. ^*^ *P*<0.05 vs. sham group. ^**^ *P*<0.05 vs. MI group. ^***^ *P*<0.05 vs. bFGF group (n=5). **(C, D)** Effects of bFGF on the infarcted area and infarct size. Paraffin-embedded tissues were stained with Masson’s trichrome, and the ratio of the infarcted area to the left ventricle field was evaluated. ^*^ *P*<0.05 vs. sham group. ^**^ *P*<0.05 vs. MI group. ^***^ *P*<0.05 vs. bFGF group (n=5).

Echocardiography indicated an increased ejection fraction in the bFGF group (64 ± 2.4%) compared with the MI group (47 ± 4.9%) and bFGF + 2-MeOE2 group (45 ± 6.5%) after treatment for 7 and 28 days (**Figure 1**). Similar results were observed for both FS (Fractional shortening) and LVSD (Left Ventricular End Systolic Diameter). The most remarkable changes were seen in the bFGF group, while there were no differences between the MI and bFGF + 2-MeOE2 group (**Figure 1C**). Representative images of Masson’s trichrome stained-tissues are shown in **Figure 2**. Remarkable reductions in fibrosis and the infarcted area were observed in the bFGF group compared with the MI and bFGF + 2-MeOE2 groups.

### Apoptotic Resistance Induced by bFGF Is Dependent on HIF-1α

**Figure 3:**
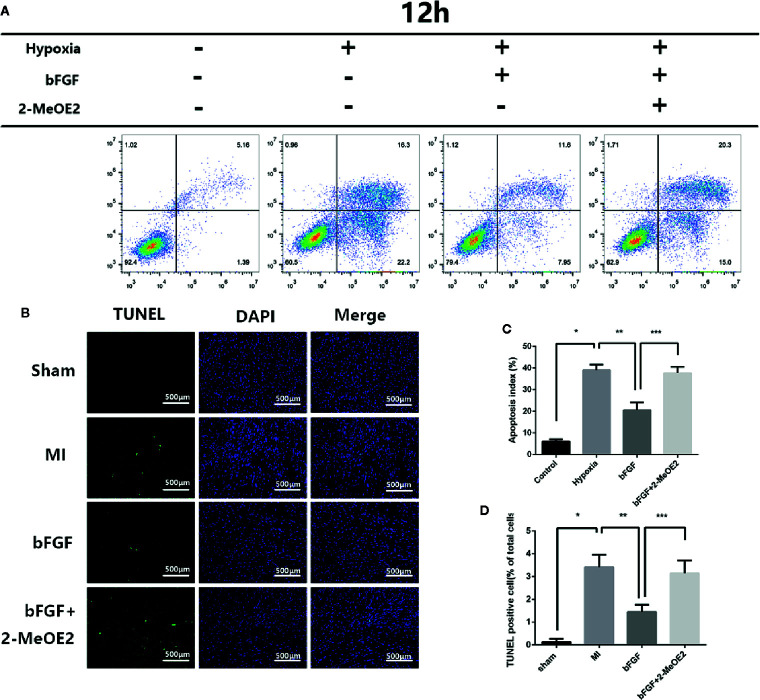
Resistance to apoptosis in infarcted areas and MMECs after treatment with bFGF. **(A)** Apoptosis was evaluated by flow cytometry. Pretreated cells were collected, stained with Annexin-V and PI, and analyzed by FCM (flow cytometry). **(C)** Apoptotic index values of the different groups. ^*^ *P*<0.05 vs. NC group; ^**^ *P*<0.05 vs. hypoxia group; ^***^ *P*<0.05 vs. hypoxia + bFGF group. ;NS, non significance vs. bFGF+2-MeOE2 group. **(B)** Evaluation of the apoptotic rate by TUNEL staining. Representative images of apoptotic cells are shown. The apoptotic cells were detected by TUNEL staining, and the nuclei were detected by DAPI. Scale bar: 20 μm. **(D)** Proportions of TUNEL-positive cells (% of total cells). ^*^ *P*<0.05 vs. MI group; ^**^ *P*<0.05 vs. bFGF group; ^***^ *P*<0.05 vs. bFGF + 2-MeOE2 group (n=3 each group).

To evaluate the cardioprotective effects of bFGF, TUNEL staining was performed at 7 days post-surgery (**Figure 3**). The numbers of TUNEL-positive nuclei in the ischemic area at 7 days were higher in the MI group and bFGF + 2-MeOE2 groups compared with the bFGF group and in the bFGF group compared with the sham group; TUNEL-positive cells were barely detected in the non-infarcted area (**Figure 3B**). Apoptosis of MMECs induced by hypoxia (37°C, 1% O_2_, N_2_, and 5% CO_2_, 12 h) was measured by flow cytometry. The proportion of apoptotic cells was lower in the bFGF group than in MMECs, and treatment with 2-MeOE2 increased the percentage of apoptotic cells (**Figure 3A**). Thus, bFGF treatment of MMECs induced an antiapoptotic effect mediated by HIF-1α.

We also examined the changes of Bax/Bcl-2 in MMECs and murine hearts. At 14 days after MI and 12 h of hypoxic incubation, the level of Bax and the ratio of Bax/Bcl-2 were downregulated in the bFGF group compared with the MI and hypoxia groups (**Figures 5C, H** and **6C, H**).

### Enhancement of Angiogenesis by bFGF

**Figure 4:**
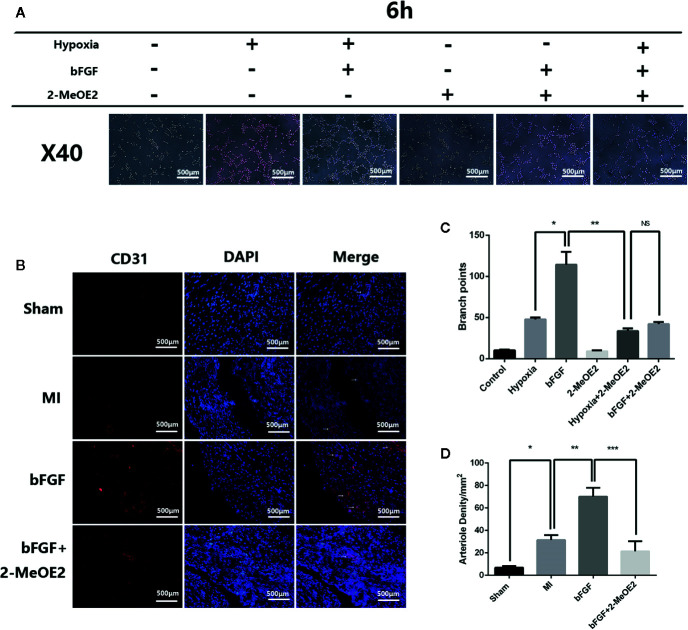
Enhanced angiogenesis in infarcted areas and MMECs after treatment with bFGF. **(A)** Effects of bFGF on tube formation of MMECs. Serum-starved MMECs were treated with or without bFGF or 2-MeOE2 for 12 h before seeding onto Matrigel. Tube formation was detected, and photos were taken at 6 h after seeding. The total length of the tubes was measured. There was greater cord formation in bFGF-treated cells (original magnification, ×40), and less cord formation in 2-MeOE2-treated cells, compared with control cells. **(C)** Tube branch points. ^*^ *P*<0.05 vs. hypoxia group; ^**^ *P*<0.05 vs. hypoxia + 2-MeOE2 group; NS vs. bFGF + 2-MeOE2 group. NS, *P*>0.05. **(B)** The left ventricle tissue was frozen, sectioned, and immunostained with anti-CD31 antibody. Violet staining indicates CD31^+^ immunofluorescent capillary and blood vessels (white arrowheads). **(D)** Arteriole densities (/mm^2^). ^*^ *P*<0.05 vs. MI group; ^**^ *P*<0.05 vs. bFGF group; ^***^ *P*<0.05 vs. bFGF + 2-MeOE2 group (n= 3 each group).

The results of immunohistochemical analyses indicated an increase in the number of cells positive for the endothelial cell marker CD31 after treatment with bFGF (**Figure 4B**). The capillary density was determined by immunofluorescence staining for CD31, which suggested increased neovascularization in the injury border area in the bFGF group compared with the MI and bFGF + 2-MeOE2 groups. These results indicated that bFGF contributes to MMEC proliferation in infarcted tissue (**Figure 4D**).

To examine whether bFGF treatment triggers tubulogenesis, we performed *in vitro* Matrigel assays using MMECs from the normal DMEM, hypoxia, hypoxia + bFGF, normal DMEM + 2-MeOE2, hypoxia + 2-MeOE2, or bFGF + 2-MeOE2 groups to analyze the extent of tube formation. The formation of capillary-like structures was significantly increased in MMECs exposed to bFGF compared with the other conditions examined. In addition, the degree of tube formation was lower in the groups treated with 2-MeOE2 than in the untreated groups. Tube formation was quantified by microscopy and showed more branch points in bFGF-treated cells than in the other treatment groups (**Figures 4A, C**).

### Activation of the p-AKT/HIF-1α/VEGF Pathway in MMECs

**Figure 5:**
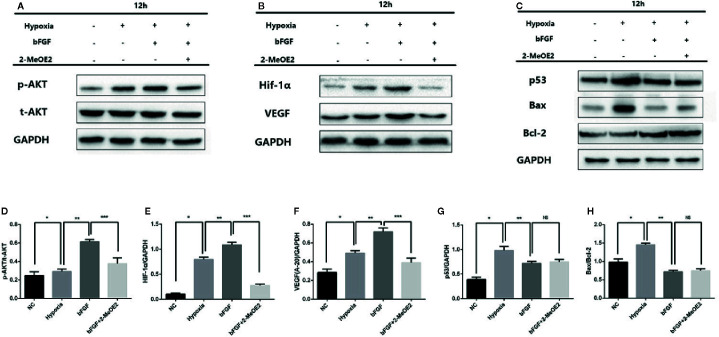
Enhanced AKT/HIF-1α pathway and reduced apoptosis-related protein levels in the MI mouse model after bFGF treatment. **(A–C)**, Effects of bFGF on p-AKT/total-AKT, HIF-1α, VEGF, p53, and Bax/Bcl-2 expression at 12 h after hypoxia. **(D–F)**, Hypoxic MMECs at 12 h after bFGF administration had higher levels of p-AKT, HIF-1α, and VEGF expression, whereas 2-MeOE2 blocked these effects of bFGF. **(G, H)**, MMECs treated with bFGF showed decreased expression of apoptosis-related cytokines (p53, Bax, Bcl-2) (n=5 each group at 14 days). ^*^ *P*<0.05 vs. sham group; ^**^ *P*<0.05 vs. MI group; ^***^ *P*<0.05 vs. bFGF group; NS, *P*>0.05.

**Figure 6:**
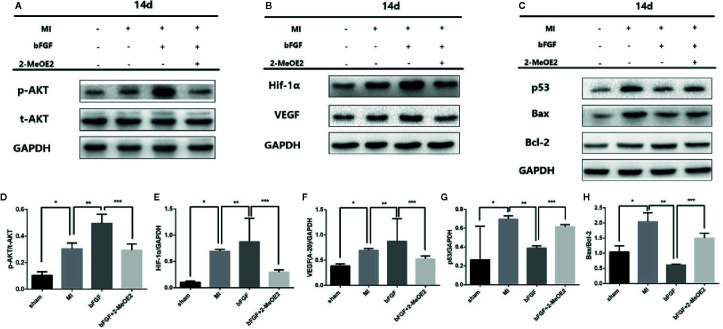
Enhanced AKT/HIF-1α pathway and reduced apoptosis-related proteins under conditions of 1% hypoxia with bFGF treatment. **(A–C)**, Effects of bFGF on p-AKT/total-AKT, HIF-1α, VEGF, p53, and Bax/Bcl-2 expression at 14 days after myocardial infarction. **(D–F)**, MI mice with 14 days of bFGF administration showed higher p-AKT, HIF-1α, and VEGF expression, whereas 2-MeOE2 blocked the effect of bFGF. **(G, H)**, MMECs by bFGF treatment decreased the expression of apoptosis-related cytokines (p53, Bax/Bcl-2). 2-MeOE2 treated mice showed increased expression of apoptosis-related cytokines (n=5 each group at 14 days). ^*^ *P*<0.05 vs. sham group; ^**^ *P*<0.05 vs. MI group; ^***^ *P*<0.05 vs. bFGF group; NS, P>0.05.

We examined the possible involvement of HIF-1α-dependent cell proliferation and antiapoptotic effects in MMECs. AKT was reported to also play a significant role in this mechanism. Phosphorylation of AKT was shown to regulate the expression of VEGF (Schultz et al., 1999) *via* HIF-1α transactivation (Sullivan et al., 2002) under conditions of ischemia. AKT increased the accumulation of HIF-1α, *via* stabilization, after hypoxia (Karni et al., 2002). Therefore, HIF-1α may mediate FGFR/PI3K/p-AKT-induced angiogenesis. At 14 days after MI and 12 h of hypoxic incubation, phosphorylation of AKT was upregulated in the bFGF group compared with the MI and hypoxia groups (**Figures 5** and **6**). The accumulation of HIF-1α was greater in MMECs exposed to hypoxia than in untreated MMECs, and this accumulation was inhibited by 2-MeOE2 (**Figure 5B**).

We analyzed the phosphorylation levels of AKT, AKT, HIF-1α, and VEGF in the murine heart in the sham, MI, bFGF, and bFGF + 2-MeOE2 groups (**Figures 6A, B**). In the infarcted area at 14 days post-surgery, the levels of AKT phosphorylation were markedly upregulated in bFGF-treated mice compared with the sham group. In addition, HIF-1α levels were higher in bFGF-treated mice than in the sham group. As 2-MeOE2 inhibited the accumulation of HIF-1α in MMECs, these findings indicate that the AKT/HIF-1α/VEGF pathway is involved in the increased VEGF production as observed (**Figures 5** and **6**).

Furthermore, we analyzed the levels of p53 in the murine heart in the sham, MI, bFGF, and bFGF + 2-MeOE2 groups (**Figures 6C, G**). P53 is a significant regulator in the induction of maladaptive hypertrophy, and at the same time, does so by inhibiting the angiogenesis of the cardiac (Sano et al., 2007). In the infarcted area at 14 days post-surgery, the levels of p53 were markedly downregulated in bFGF-treated mice compared with the MI group (**Figure 6G**).

## Discussion

Ischemia has detrimental effects on the function and structure of the myocardium. During the early stages of coronary atherosclerosis, the initial cardiac responses to hypoxia may serve an organ protective effect. Maladaptation or chronic insults will lead to cardiac fibrosis, which may lead to heart failure. This theory prompted us to assess the relationship between bFGF and cardioprotection and whether HIF-1α accumulation can have a role in mediating FGF2 promotion of angiogenesis and antiapoptosis in cardiac endothelial cells.

Mice were treated with constitutively active bFGF, and the results indicated that continuous activation of bFGF enhanced AKT/HIF-1α/VEGF-dependent angiogenesis/survival of MMECs, leading to changes in cardiac function related to mature neovascularization and reduced cardiac remodeling. We found that bFGF was positively related to heart recovery in bFGF-treated models.

We detected two crucial proteins (HIF-1α and VEGF) regulated during cardioprotection at 14 days of treatment with bFGF. At 2 weeks of continuous bFGF treatment, the HIF-1α level was markedly increased in the bFGF group compared with the other groups and the protective effect of bFGF was markedly blocked by HIF-1α inhibitor 2-MeOE2. HIF-1α was induced by bFGF, according to the significant increase in its expression after inhibiting the degradation of this transcription factor. Consequently, the level of VEGF, a crucial downstream growth factor, was significantly increased, which resulted in enhanced angiogenesis. Interestingly, HIF-1α and VEGF were also increased in the MI group. It was suggested that the effects of hypoxia could be reversed to some extent, as HIF-1α degradation increased, thereby returning HIF-1α and downstream VEGF to relatively low levels (Sheikh et al., 2001).

Moreover, no remarkable differences were found between the 2-MeOE2 and MI groups in the expression levels of HIF-1α, indicating that accumulation of HIF-1α is crucial for bFGF to promote angiogenesis and antiapoptosis and to protect injured cardiomyocytes. HIF-1α was reported to be degraded in normoxia because of the ubiquitination of oxygen-dependent degradation (ODD) domain (Xiao et al., 2015). bFGF may be involved in this crucial kinetic pathway of the HIF-1α. At the same time, there is a doubt that the MMEC proliferation of group Hypoxia +2MeOE2 higher than group 2MeOE2 even though the activation of Hif-1a is cancelled by 2MeOE2. We have a guess that including Hif-1a there are other pathways to promote capillary formation when the Hif-1a pathway was blocked. Silent information regulator protein 1 (SIRT1) may be a potential mechanism (Li et al., 2018).

In summary, bFGF treatment resulted in reduced infarct size and changes in cardiac function related to antiapoptotic effects and enhanced mature neovascularization. AKT-dependent survival and migration of MMECs were significantly activated by bFGF, and bFGF treatment increased AKT/HIF1α/VEGF activation and antiapoptotic actions in the myocardium and improved peri-vessel migration of MMECs, leading to enhanced bFGF-mediated cardioprotection. Therefore, our results suggest that the specific cardioprotective action of bFGF may be attributable to the enhanced angiogenic activities by the activated AKT/HIF-1/VEGF pathway.

It was reported that there was no remarkable loss of cardiac function when blood pressure was decreased in FGF2 knockout mice; however, a crucial reduction in cardiac hypertrophy was observed with aortic coarctation (Masoud and Li, 2015). This suggested that FGF2 plays a significant role in cardiac growth in response to hemodynamic load. Meanwhile, targeted disruption of the FGF2 gene had no effect on vascular growth in a hind limb ischemia model (Schultz et al., 1999). In response to ischemia–reperfusion injury in mice, FGF2 overexpression in a cardiac-specific manner resulted in a cardioprotective effect with increased angiogenic and vascular functions affecting the infarct size (Sullivan et al., 2002). Furthermore, FGF2-overexpressing mice showed attenuation of the damage related to ischemic injury by increasing the capillary density in the infarct border area (Karni et al., 2002). These studies have promoted a great deal of interest in the therapeutic strategy of inducing angiogenesis *via* bFGF (Post et al., 2001). Our results presented here suggest an additional mechanism that the accumulation of HIF-1α promoted by bFGF can also enhance angiogenesis and antiapoptotic effects in cardiac endothelial cells and tissues, thereby protecting the ischemic myocardium. However, further independent investigation in a HIF-1α gene knockout mouse model is required to determine the significance of accumulation of HIF-1α in mediating the FGF-induced angiogenesis and antiapoptotic effects.

## Conclusion

We demonstrated that the accumulation of HIF-1α under bFGF treatment can enhance angiogenesis and antiapoptotic effects in cardiac endothelial cells, as well as protect the ischemic myocardium. Our findings suggest that a novel mechanism by which bFGF attenuates the injury induced by myocardial infarction by enhancing the accumulation of HIF-1α.

## Data Availability Statement

The raw data supporting the conclusions of this article will be made available by the authors, without undue reservation.

## Ethics Statement

The animal study was reviewed and approved by the Ethics Committee for Experimental Animals of Wenzhou Medical University.

## Author Contributions

ZR performed research, analyzed data and wrote the paper. DS performed research and contribute reagents. JC performed research and analyzed data. LJ performed research. XW analyzed data. MiC analyzed data. LL designed research. MaC designed research. JL designed research and wrote the paper.

## Funding

This work was supported by grant 81570299, grant 81570238, grant 81570342 and grant 81870281 from the National Natural Science Foundation of China and WKJ-ZJ-1725 from the Technology Plan Project of Zhejiang Province and Y20160027 from the Foundation of Wenzhou science and technology bureau.

## Conflict of Interest

The authors declare that the research was conducted in the absence of any commercial or financial relationships that could be construed as a potential conflict of interest.
